# A chromosome-level reference genome of the hornbeam, *Carpinus fangiana*

**DOI:** 10.1038/s41597-020-0370-5

**Published:** 2020-01-21

**Authors:** Xiaoyue Yang, Zefu Wang, Lei Zhang, Guoqian Hao, Jianquan Liu, Yongzhi Yang

**Affiliations:** 1 Stat Key Laboratory of Grassland Agro-Ecosystem, Institute of Innovation Ecology, Lanzhou University, 730000 Lanzhou, China; 2 Stat Key Laboratory of Bio-Resource and Eco-Environment of Ministry of Education, College of Life Sciences, Sichuan University, Chengdu, 610000 Sichuan China; 3 Sichuan Tea College, Yibin University, Yibin, 644007 Sichuan China

**Keywords:** Genome, Genomics

## Abstract

Betulaceae, the birch family, comprises six living genera and over 160 species, many of which are economically valuable. To deepen our knowledge of Betulaceae species, we have sequenced the genome of a hornbeam, *Carpinus fangiana*, which belongs to the most species-rich genus of the Betulaceae subfamily Coryloideae. Based on over 75 Gb (~200x) of high-quality next-generation sequencing data, we assembled a 386.19 Mb *C. fangiana* genome with contig N50 and scaffold N50 sizes of 35.32 kb and 1.91 Mb, respectively. Furthermore, 357.84 Mb of the genome was anchored to eight chromosomes using over 50 Gb (~130x) Hi-C sequencing data. Transcriptomes representing six tissues were sequenced to facilitate gene annotation, and over 5.50 Gb high-quality data were generated for each tissue. The structural annotation identified a total of 27,381 protein-coding genes in the assembled genome, of which 94.36% were functionally annotated. Additionally, 4,440 non-coding genes were predicted.

## Abstract (summary)

**Table:** 

Measurement(s)	DNA • RNA • sequence_assembly • sequence feature annotation
Technology Type(s)	DNA sequencing • RNA sequencing • genome assembly • sequence annotation
Sample Characteristic - Organism	Carpinus fangiana

Machine-accessible metadata file describing the reported data: 10.6084/m9.figshare.11382390

## Background & Summary

Betulaceae, also known as the birch family, includes over 160 species of trees or shrubs^1^. It is divided into two subfamilies, Coryloideae and Betuloideae; Betuloideae comprises the genera *Alnus* and *Betula*, while Coryloideae comprises *Corylus*, *Ostryopsis*, *Carpinus* and *Ostrya*. These subfamilies and their genera are readily distinguished based on their different morphological characteristics, such as the samara of Coryloideae, the nuts of Betuloideae, and their different types of pollen^2^. In addition, cell biological investigations have revealed that Betulaceae species have very different chromosome numbers: the basic chromosome number is eight for *Carpinus*, *Ostrya*, *Ostryopsis* species, eleven for *Corylus* species, and fourteen for *Alnus* and *Betula* species^3,4^.

Several Betulaceae species, notably those belonging to the genera *Betula*, *Alnus*, and *Carpinus*, are important components of forests in temperate regions, mountains, and subtropical areas, as well as important sources of timber and materials for traditional Chinese medicine. Some species of *Betula* and *Carpinus* are used as ornamental trees and widely planted in large parks and gardens. *Alnus* species can form symbioses with nitrogen-fixing bacteria of the genus *Frankia*, helping to enhance soil fertility^5^. The fruits of *Corylus*, known as hazelnuts, are economically important. The birch family thus has remarkable ecological, economic, medicinal, and ornamental value. Additionally, Betulaceae is a relict family, and there are many reliable fossils of this family that have provided important paleobotanic insights^6^. However, only a few species of the family have been studied extensively in ways that could support their further development and utilization.

A few genomes of Betulaceae species have been published in recent years. The genomes of two Betuloideae members, *Betula pendula* (scaffold N50: 0.53 Mb)^7^ and *Alnus glutinosa* (scaffold N50: 0.10 Mb)^8^, were presented in 2017 and 2018, and the *B. pendula* genome was further anchored to fourteen chromosomes. The only published Coryloideae genomes are those of two ironwood trees from the genus *Ostrya*: *O*. *rehderiana* (scaffold N50: 2.31 Mb) and *O. chinensis* (scaffold N50: 0.81 Mb), which were reported in 2018^9^. However, no genomes representing any of the other three genera in Coryloideae have been disclosed and there are no published chromosome-level genomes for this subfamily.

**Fig. 1:**
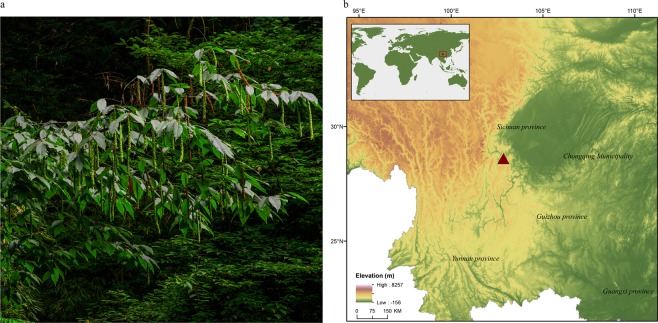
Photograph and location of the *C. fangiana* tree sampled for genome sequencing. (**a**) A photograph of a *C. fangiana* individual on Emei Mountain, Leshan, Sichuan, China. (**b**) Location of the *C. fangiana* sample used for genome sequencing.

To enrich the available genomic resources for Betulaceae, we sequenced the whole genome of *Carpinus fangiana* (Fig. 1), a member of the most species-rich genus in Coryloideae^10^. A total of 77.85 Gb (~200x) next-generation data and 52.19 Gb (~130x) Hi-C data were used to assemble the genome. The assembly produced a genome having a total length of 386.19 Mb, with 357.84 Mb being anchored to eight chromosomes. To our knowledge, this is the first reported chromosome-level Coryloideae genome assembly. The contig N50 and scaffold N50 were 35.32 kb and 1.91 Mb, respectively. Structural annotation of the genome revealed a total of 27,381 protein-coding genes, of which 94.36% were functionally annotated. The genome was also predicted to contain 4,440 non-coding genes based on a comprehensive annotation. This chromosome-level genome of *C. fangiana* will greatly facilitate further biological studies on Betulaceae as well as the development and commercial exploitation of the genus.

## Methods

### Sampling, library construction and sequencing

Fresh leaves were collected from a wild *C. fangiana* tree in Ebian, Sichuan, China (N: 29° 1′44″; S: 102°59′30″; Fig. 1) and immediately dried over silica gel. Genomic DNA was then extracted from the dried leaves using the modified Cetyltrimethylammonium Ammonium Bromide (CTAB)^11^ method. Sequencing libraries with different insert sizes were constructed using a library construction kit (Illumina). Short paired-end libraries were constructed with insert sizes of 230, 500, and 800 bp, while the insert sizes used to construct mate pair libraries were 2, 5, 10, and 20 kb. The Illumina HiSeq 2000 platform was used to sequence 150 bp paired-end reads for all these libraries in accordance with the manufacturer’s instructions. These procedures generated a total of 115.12 Gb (~200x) raw data for *C. fangiana* genome assembly (Table 1).

**Table 1:** DNA sequencing metrics of *C. fangiana*, before and after quality control.

Sequencing technique	Library type	Insert size (bp)	Read length (bp)	Amount of sequence		Depth (x-times)	
				Raw data (Gb)	clean data (Gb)	Raw data	clean data
Next-generation	paired-end	230	150	11.32	10.92	28.54	27.52
	paired-end	500	150	10.28	10.21	25.91	25.73
	paired-end	800	150	15.82	15.64	39.88	39.42
	mate pair	2,000	150	16.49	6.55	41.56	16.51
	mate pair	5,000	150	13.25	9.71	33.39	24.47
	mate pair	10,000	150	17.97	10.71	45.30	27.00
	mate pair	20,000	150	29.99	14.12	75.59	35.59
	Total			115.12	77.85	290.17	196.23
Hi-C	Hi-C	300-700	150	52.54	52.19	132.43	131.55

Note: The data contains Next-generation and Hi-C sequencing data. The estimated genome size is 396.74 Mb.

A High-through chromosome conformation capture (Hi-C) library for the *C. fangiana* genome was also constructed. To this end, fresh leaves were fixed with formaldehyde to induce DNA cross-linking, after which the DNA was digested with HindIII. The resulting sticky ends were biotinylated and proximity-ligated to form chimeric junctions that were enriched for, and physically sheared into 300–700 bp fragments. These chimeric fragments were sequenced on the Illumina HiSeq platform, generating 52.54 Gb (~130x) of Hi-C data (Table 1).

We also harvested six tissues (bark, branch, bract, flower, fruit, leaf) for total RNA sequencing. These samples were flash frozen in liquid nitrogen, and total RNA was extracted using the modified CTAB method^12^. cDNA libraries were then constructed using the NEBNext Ultra RNA Library Prep Kit for Illumina (NEB). The Illumina HiSeq 2500 platform was used to sequence these libraries with a read length of 2 × 150 bp, generating over 5.50 Gb raw data for each tissue (Table 2).

**Table 2:** Illumina RNA sequencing metrics, before and after quality control.

Tissue	Raw reads	Clean reads	Raw bases (Gb)	Clean bases (Gb)
Bark	19,815,362	19,725,663	5.95	5.92
Branch	22,825,277	22,766,831	6.85	6.83
Bract	22,847,208	22,789,778	6.85	6.84
Flower	34,835,605	34,834,910	10.45	10.45
Fruit	18,628,078	18,570,700	5.59	5.57
Leaf	21,888,088	22,789,778	6.57	6.55

### Preprocessing and genome size estimation

Quality control checks on the raw genome data were preformed using FastQC (http://www.bioinformatics.babraham.ac.uk/projects/fastqc/). Potential adapters in reads were removed using Scythe (http://github.com/vsbuffalo/scythe) and low-quality reads were discarded by Sickle (http://github.com/vsbuffalo/scythe). The program Lighter^13^ was then used to correct sequence errors in the remaining reads. For mate pair reads, we also used FastUniq^14^ to remove duplicates. In total, 77.85 Gb, ~200x high-quality next-generation sequencing data and 52.19 Gb, ~130x high-quality Hi-C data were generated for *de novo* assembly of the *C. fangiana* genome (Table 1).

Quality control of transcriptome data was performed using a custom Perl script. Reads were discarded if (1) the proportion of unidentified nucleotides in one read exceeded 5%, or (2) over 65% of the read’s bases had a phred quality below 8. After eliminating low-quality reads, the quantity of retained data for each tissue was above 5.50 Gb (Table 2). The RNA-seq reads were then assembled using Trinity^15^. CD-Hit^16^ was used to eliminate redundant transcript sequences, and candidate coding regions in the transcript sequences were identified by TransDecoder (https://transdecoder.github.io).

**Fig. 2:**
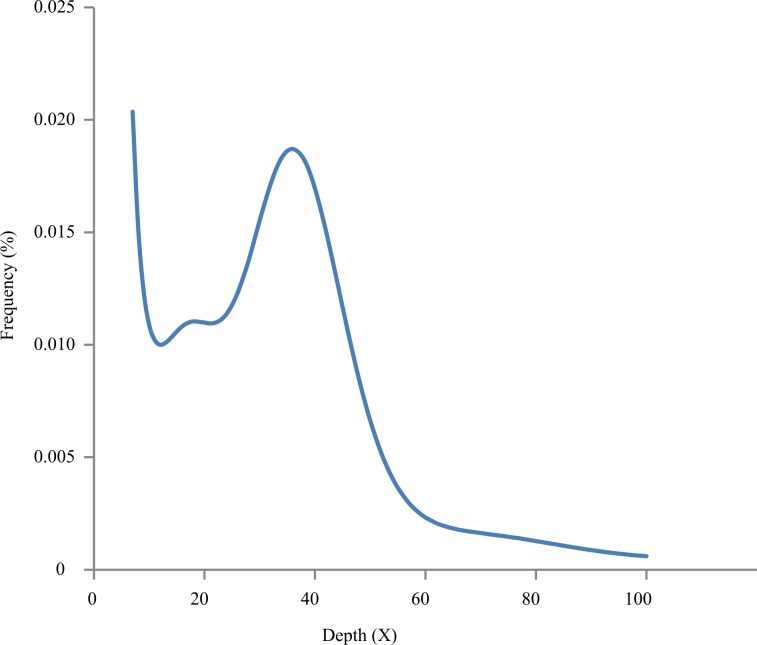
K-mer distribution used to estimate the genome’s size. The distribution was determined based on the Jellyfish analysis using a *k*-mer size of 17.

Before genome assembly, we estimated the *C. fangiana* genome’s size by performing a combined analysis using Jellyfish^17^ and GenomeScope^18^. Reads from the short-insert libraries were first processed by Jellyfish to assess their *k*-mer distribution, using a *k* value of 17. Then, GenomeScope was used to estimate the genome size based on the *k*-mer distribution (Fig. 2). The genome was thereby estimated to be around 396.74 Mb long.

### Genome assembly

Preliminary *de novo* assembly of the *C. fangiana* genome was performed with Platanus^19^, which can effectively manage high-throughput data from heterozygous samples. Assembly using Platanus proceeded via three steps: (1) contig-assembly, in which de Bruijn graphs were constructed using the clean reads from short paired-end libraries and the sequences of contigs were then displayed in the graphs; (2) scaffolding, in which reads from all next-generation libraries (short paired-end and mate pair) were mapped to contigs, after which contigs considered to be linked were combined into scaffolds; (3) gap closing, in which reads that mapped onto scaffolds were collected to cover the gaps between them. GapCloser^20^ was used to further close the gaps based on reads from all the paired-end libraries, after which the automated HaploMerger2 pipeline^21^ was used to rebuild the above assembly and implement flexible and sensitive error detection. After discarding scaffolds smaller than 1 kb, a high-quality *de novo* assembled *C. fangiana* genome was obtained. The size of this genome (386.19 Mb) was 97.34% of the estimated value (396.74 Mb) and its GC content was 37.59%. The scaffold N50 and N90 values were 1.91 Mb and 0.43 Mb, while the contig N50 and N90 were 35.32 kb and 8.54 kb (Table 3).

**Table 3:** Summary of *C. fangiana* genome assembly.

Type	*De novo* assembly	Hi-C assembly
Scaffold length (bp)	386,190,506	386,249,499
Gap length (bp)	30,727,985	30,804,875
Scaffold number	4,789	4,602
Longest scaffold (bp)	8,871,445	60,187,804
Scaffold N50 (bp)	1,908,393	37,105,143
Scaffold N90 (bp)	425,779	595,656
Contig length (bp)	355,461,404	355,441,862
Contig number	21,775	22,086
Longest contig (bp)	1,041,408	912,918
Contig N50 (bp)	35,323	34,845
Contig N90 (bp)	8,542	8,427
GC content	37.59%	37.55%

Note: The estimated genome size is 396.74 Mb. GC content of the genome without N.

The HiC-Pro^22^ program was used for quality assessment of the Hi-C data. Valid interaction pairs were mapped to and used for error correction of the contigs and scaffolds assembled based on the next-generation sequencing data. Next, the contigs and scaffolds were anchored to chromosomes using LACHESIS^23^. In total, 357.84 Mb of scaffolds were assembled into eight chromosomes (Table 4). Finally, we obtained a high-quality chromosome-level genome with a total size of 386.25 Mb. The contig N50 and scaffold N50 values of this chromosome-level assembly were 34.85 kb and 37.11 Mb, respectively (Table 3).

**Table 4:** Summary of the assembled chromosomes in the *C. fangiana* genome.

Type	Sequence Number	Sequence Length (bp)	GenBank accession
Cfa01	128	62,383,991	CM017321
Cfa02	97	51,103,020	CM017322
Cfa03	107	42,654,226	CM017323
Cfa04	135	44,816,785	CM017324
Cfa05	88	39,651,540	CM017325
Cfa06	104	40,118,261	CM017326
Cfa07	92	39,687,453	CM017327
Cfa08	109	37,421,582	CM017328
Total Sequences Clustered (Ratio %)	860 (16.32)	357,836,858 (92.66)	
Total Sequences Ordered and Oriented (Ratio %)	677 (78.72)	319,127,541 (89.18)	

### Heterozygosity assessment and repeat annotation

To assess the heterozygosity of the *C. fangiana* genome, we first mapped reads from the 500 bp library to the assembled genome using the BWA-MEM algorithm from the Burrows-Wheeler Aligner (BWA) package^24^. SAMtools^25^ was used to convert the mapping results to BAM format, sort them, and remove duplicates. The Picard package (http://broadinstitute.github.io/picard/) was used to replace read groups in the bam file. Two programs (RealignerTargetCreator and IndelRealigner) from the Genome Analysis ToolKit (GATK)^26^ package were used to avoid misalignments and account for the effects of indels. The SAMtools command ‘mpileup’ was used to generate a VCF format file, and the program bcftools from the SAMtools package was used to detect single nucleotide polymorphisms (SNPs). Finally, based on the SNPs, the heterozygosity was calculated to be 0.38% using a custom Perl script.

Repetitive sequences and transposable elements (TEs) in the *C. fangiana* genome were identified using a combined procedure incorporating *de novo* and homology-based approaches at the DNA and protein levels. Tandem repeats were annotated using Tandem Repeat Finder (TRF)^27^. A repeat library for the *C. fangiana* genome was generated using RepeatModeler (http://www.repeatmasker.org) to facilitate *de novo* annotation. RepeatMasker^28^ (http://www.repeatmasker.org) was used to identify and classify the TEs at the DNA level. We also used RepeatProteinMasker to perform a WU-BLASTX search against the TE protein database in order to identify and classify TEs at the protein level. Finally, long terminal repeats (LTR) were identified using LTR-FINDER^29^. In total, the *C. fangiana* genome was found to contain 158.69 Mb repetitive sequences, accounting for 41.08% of its length (Table 5). As shown in Table 5, the most common classifications assigned to these repetitive elements were Unknown (15.97% of the assembled genome) and LTRs (14.57% of the assembled genome).

**Table 5:** Repeat element metrics for the *C. fangiana* genome.

Type	Length (bp)	Percent (%)
DNA	14,244,548	3.69
LINE	15,452,667	4.00
Low_complexity	1,653,498	0.43
LTR	56,262,090	14.57
Other	660	1.71E-04
RC	1,272,200	0.33
rRNA	5,881	1.52E-03
Satellite	232,066	0.06
Simple_repeat	7,594,441	1.97
SINE	281,915	0.07
Uknown	61,686,663	15.97
All	158,686,629	41.08

### Gene annotation

Structural annotation of gene models was performed by applying a combination of *de novo*, homology-based, and transcriptome-based methods to the repeat-masked genome. The *de novo* approach was implemented using Augustus^30^, Geneid^31^, GeneMark^32^, glimmerHMM^33^, and SNAP^34^. For homology-based prediction, TBLASTN^35^ was used to align predicted protein sequences from *Arabidopsis thaliana*, *Vitis vinifera*, *Prunus persica*, *Ostrya chinensis*, *Ostrya rehderiana* and *Juglans regia* to the *C. fangiana* genome with an E-value threshold of 1E-05. Then, GeneWise^36^ was used to obtain accurate spliced alignments by aligning homologous sequences to matched proteins. Transcriptome-based prediction was performed with the Program to Assemble Spliced Alignments (PASA)^37^, which was used to predict protein-coding regions based on the assembled transcripts of the six different *C. fangiana* tissues. The gene models obtained from the *de novo*, homology-based, and transcriptome-based annotations were combined to form a consensus gene set using EVidenceModeler (EVM)^38^. After strict filtering, a total of 27,381 non-redundant protein-coding genes were annotated in the *C. fangiana* genome (Table 6).

**Table 6:** Summary of predicted protein-coding genes in the *C. fangiana* genome.

Gene set		Number	Average gene length (bp)	Average CDS length (bp)	Average exons per gene	Average exon length (bp)	Average intron length (bp)
*De novo* prediction	Augustus	36,499	3,740.33	1,371.15	5.20	342.17	678.20
	Geneid	43,054	4,539.67	1,023.87	4.14	247.27	1,755.27
	GeneMark	28,642	1,900.29	892.05	3.15	283.15	492.58
	GlimmerHMM	45,800	1,657.35	867.05	2.65	327.78	398.26
	SNAP	63,982	1,087.42	656.98	2.62	250.80	220.80
Homolog prediction	*Arabidopsis thaliana*	21,976	3,251.94	1,100.22	4.45	247.27	631.93
	*Vitis vinifera*	23,733	3,293.62	1,047.44	4.59	228.23	633.86
	*Prunus persica*	24,493	3,204.43	1,088.71	4.35	250.14	639.44
	*Juglans regia*	25,252	3,200.15	1,076.69	4.24	253.84	662.00
	*Ostrya rehderiana*	31,130	2,907.56	990.15	4.00	247.72	647.70
	*Ostrya chinensis*	32,669	2,901.71	958.97	3.94	243.51	668.90
RNA seq	PASA	33,115	5,076.06	1,100.55	5.09	414.69	800.10
EVM		36,585	3,692.57	1,283.06	4.67	274.71	1,197.00
PASA update*		36,439	4,067.94	1,384.96	5.27	320.73	1,253.00
Final*		27,381	3,948.29	1,415.09	5.16	345.16	1,165.54

Note: *UTR regions were contained.

Functional annotation of the predicted protein genes was performed by using BLASTP with an E-value threshold of 1E-05 to search for homologous sequences in SwissProt (http://www.gpmaw.com/html/swiss-prot.html), TrEMBL (http://www.uniprot.org)^39^, and KEGG (http://www.genome.jp/kegg/) protein databases^40^. The program hmmscan of HMMER package (http://hmmer.org) was used to search the Pfam domains. InterProScan^41^ was used to annotate the protein motifs and domains, and the Blast2GO pipeline^42^ was used to obtain Gene Ontology (GO)^43^ IDs for each gene based on the NCBI NR database. In total, 25,836 protein-coding genes, corresponding to 94.36% of the total predicted gene models in the *C. fangiana* genome were successfully functionally annotated (Table 7).

**Table 7:** Summary of functional annotation in the *C. fangiana* genome.

Type	Gene number	% in genome
Total	27,381	
GO	19,679	71.87
KEGG	18,845	68.83
InterProScan	15,582	56.91
Pfam	19,688	71.90
Uniprot_sprot	19,733	72.07
Uniprot_trembl	24,110	88.05
All	25,836	94.36

We also annotated non-coding RNAs in the *C. fangiana* genome. tRNAscan-SE^44^ was used to detect putative transfer RNAs (tRNAs) with eukaryotic parameters, resulting in the identification of 632 tRNAs. To identify other non-coding RNAs, INFERNAL^45^ was used to perform searches against the Rfam^46^ database, resulting in the identification of 936 ribosomal RNAs (rRNAs), 197 microRNAs (miRNAs), 117 small nuclear RNAs (snRNAs), and 232 small nucleolar RNAs (snoRNAs) (Table 8).

**Table 8:** Summary of non-coding genes in the *C. fangiana* genome.

Type	Number	Average length (bp)	Total length (bp)	% of genome
tRNA	632	76.71	48,478	0.01255
rRNA	936	122.70	114,844	0.03136
miRNA	197	124.27	24,481	0.00669
snRNA	117	141.58	16,565	0.00452
snoRNA	232	97.28	22,570	0.00616
SRPRNA	9	280.33	2,523	0.00069
other ncRNA	2,317	109.13	252,859	0.06905
Total	4,440	108.63	482,320	0.12490

## Data Records

The sequencing data including the Illumina genome data (SRA accession: SRX6070999-SRX6071006), Hi-C data (SRA accession: SRX6071007), and Illumina transcriptome data (SRA accession: SRX6070994-SRX6070998, SRX6071008) were submitted to the NCBI Sequence Read Archive (SRA) database under BioProject accession number PRJNA548027^47^. The assembled genome was deposited at DDJB/ENA/GenBank under accession number VIBQ00000000^48^. Repeat annotations, gene model annotations and non-coding RNA annotations, the CDS sequences for the coding and non-coding genes, the protein sequences for the coding genes, as well as two custom Perl scripts were deposited at figshare^49^.

## Technical Validation

### Assessment of the genome assembly

We evaluated the completeness of the *C. fangiana* genome assembly in two ways. First, all the paired-end reads were mapped to the assembly genome with BWA. The aligned outputs were then analyzed using SAMtools. The mapping rate for each library was above 90% (Table 9). Furthermore, the coverage of the genome after gap elimination was 99.74%, with 95.05% having at least 100x coverage. Benchmarking Universal Single-Copy Orthologs (BUSCO)^50^ was also used to evaluate the completeness of the genome assembly. 95.30% of the “complete BUSCOs” were successfully identified in the assembly, and the proportion of “missing BUSCOs” was only 4.10% (Table 10). These results demonstrate the high reliability and completeness of the reported genome assembly.

**Table 9:** Mapping ratio of Illumina DNA reads for the *C. fangiana* genome.

Reads		Genome	
Library (bp)	Mapping rate (%)	Coverage	Value (%)
230	93.19	at least 1x	99.74
500	91.04	at least 10x	99.28
800	90.54	at least 20x	98.87
2 k	99.07	at least 30x	98.87
5 k	99.42	at least 50x	98.51
10 k	98.93	at least 80x	97.84
20 k	98.36	at least 100x	95.03

**Table 10:** Assessment of BUSCOs in the *C. fangiana* genome.

BUSCOS	Number	Percent
Complete BUSCOs	1,372	95.30%
Complete and single-copy BUSCOs	1,329	92.30%
Complete and duplicated BUSCOs	43	3.00%
Fragmented BUSCOs	8	0.60%
Missing BUSCOs	60	4.10%
Total BUSCO groups searched	1,440	

**Fig. 3:**
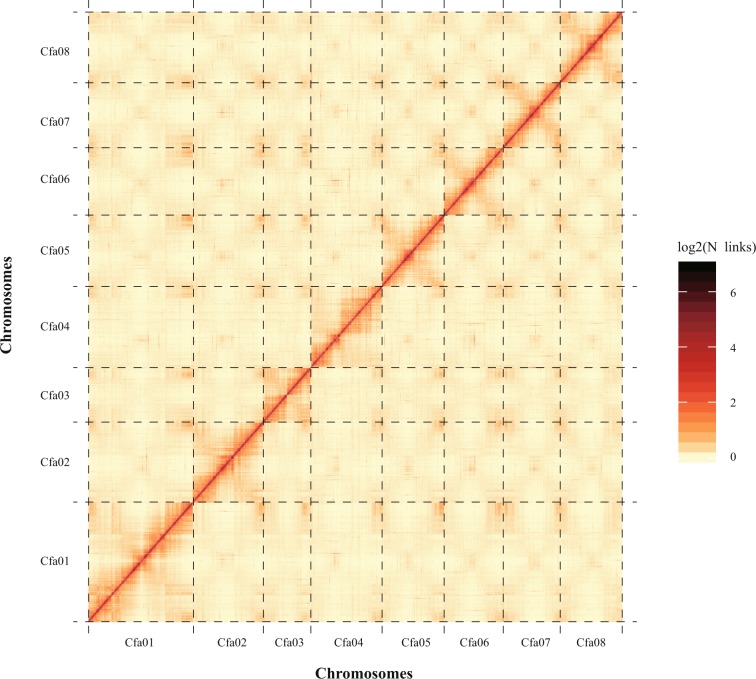
Heat map of chromosomal interactions in the *C. fangiana* genome. Cfa01-Cfa08 represent the eight chromosomes in the *C. fangiana* genome. The horizontal and vertical coordinates represent the order of each ‘bin’ on the corresponding chromosome.

Finally, we evaluated the assembly of the eight chromosomes. To this end, the anchored genome was split into ‘bins’ of 100 kb in length. The number of Hi-C read pairs covered by any two ‘bins’ was used to define the signal for the interaction between those ‘bins’, and these signal intensities were plotted in the form of a heat map. The signal intensities clearly divided the ‘bins’ into eight distinct groups, demonstrating the high quality of the chromosome assembly (Fig. 3).

### Improvement of gene annotation quality

To maximize the reliability of the gene annotation process, repeat regions in the assembled genome were masked before gene annotation. Mirroring the procedure used to filter gene annotation, EVM was initially used to merge the results obtained by *de novo*, homolog-based, and transcriptome-based predictions. Genes were then discarded if: (1) their CDS length was below 150 bp; (2) their putative coding regions could not be accurately translated into protein sequences; (3) they possessed early termination codons; or (4) they were only supported by *de novo* predictions. In addition, PASA was used to identify untranslated regions (UTRs).

## Data Availability

This work relied on many software tools. The versions, settings and parameters of these tools are given below. **(1) FastQC**: version 0.11.5, default parameters; **(2) Scythe**: version 0.994 BETA, parameters: -q sanger --quiet; **(3) Sickle**: version 1.33, parameters: pe -t sanger -q 20 -l 50 -n --quiet; **(4) Lighter**: version 1.0.7, parameters: -K 21 360000000; **(5) FastUniq**: version 1.1, default parameters **(6) Trinity**: trinityrnaseq-2.6.4, parameters: --seqType fq --JM 260G; **(6) CD-Hit**: version 4.6, default parameters; **(7) TransDecoder**: version 5.2.0, default parameters; **(8) Jellyfish**: version 1.1.10, parameters: count command: -m 17 -s 4G -c 7, dump command: -c -t, histo command: default parameters; **(9) GenomeScope**: version 2.0, parameters: 17 (*k*-mer length) 150 (read length); **(10) Platanus**: version 1.2.1, default parameters for the all three steps, **(11) GapCloser**: version 1.12, parameter: -l 150; **(12) HaploMerger2**: version HaploMerger2_20151124, default parameters for the followed running processes: carrying out batchA to batchE with the recommended pipeline, among which batchA was repeated 3 times and batchD was repeated 2 times, respectively; **(13) HiC-Pro**: version 2.10.0, default parameters; **(14) LACHESIS**: released in 2017, parameters: CLUSTER_MIN_RE_SITES=36 CLUSTER_MAX_LINK_DENSITY=1 CLUSTER_NONINFORMATIVE_RATIO=8 ORDER_MIN_N_RES_IN_TRUN=22 ORDER_MIN_N_RES_IN_SHREDS 22; **(15) BWA**: version 0.7.12-r1039, default parameters; **(16) SAMtools**: version 1.5, parameters: view command: -bS, sort command: -O BAM, depth command: -Q 40, mpileup command: -DSug -C 50, default parameters for the rmdup, index and flagstat commands; **(17) Picard**: version 1.80, parameters: SORT_ORDER =coordinate RGPL =illumina RGPU =illumina; **(18) GATK**: version 3.3-0-g37228af, default parameters for the two programs RealignerTargetCreator and IndelRealigner; **(19) bcftools**: version 0.1.19-44428 cd, parameters: view –Ncg; **(20) TRF**: version 4.07b, parameters: Match=2 Mismatch=7 Delta=7 PM=80 PI=10 Minscore=50 MaxPeriod=500 -d –h; **(21) RepeatModeler**: version 1.0.4, parameters: -pa 30 -database Fan; **(22) RepeatMasker**: version open-4.0.5, parameters: -pa 30 -species all -nolow -norna -no_is -gff; **(23) RepeatProteinMasker**: version 2.1, parameters: -engine abblast -noLowSimple -pvalue 1e-04; **(24) LTR-FINDER**: version 1.05, default parameters; **(25) Augustus**: version 2.5.5, parameters: --species=arabidopsis; **(26) Geneid**: version 1.4, parameters: -3 -P; **(27) GeneMark**: version 3.47, parameters: -f gff3; **(28) GlimmerHMM**: version 3.0.4, default parameters; **(29) SNAP**: version 2006-07-28, default parameters; **(29) GeneWise**: version 2.4.1, parameters: -tfor/-trev -gff; **(30) EVM**: version 1.1.1, default parameters; **(31) PASA**: version 2.0.2, parameters: for Launch_PASA_pipeline.pl step: -C -R -r–ALIGNERS blat, gmap, default parameters for the below two steps: asa_asmbls_to_training_set.extract_reference_orfs.pl and pasa_asmbls_to_training_set.dbi; **(32) BLASTP**: version 2.2.30+, parameters: -evalue 1e-5 -outfmt 7; **(33) Interproscan**: version 5.25-64.0, parameters: -dp -f tsv; **(34) tRNAscan-SE**: tRNAscan-SE-2.0, default parameters; **(35) BUSCO**: version 2.0, parameters: -m genome -c 20.
